# Supplementary material for: Association of hypertensive disorder of pregnancy with necrotizing enterocolitis in very preterm infants: A retrospective cohort study
Source: PLoS One. 2024 Nov 11;19(11):e0313035. doi: 10.1371/journal.pone.0313035 (PMC11554212; doi:10.1371/journal.pone.0313035)
Supplement: S1 Table — a adjusted for Birth weight, Antenatal steroids, MgSO4, Chorioamnionitis, Breast feeding, PDA. b adjusted for Gestational age, Antenatal steroids, MgSO4, Chorioamnionitis, Breast feeding, PDA. c adjusted for Antenatal steroids, MgSO4, Chorioamnionitis, Breast feeding, PDA. d adjusted for Birth weight, Gestational age, Antenatal steroids, MgSO4, Chorioamnionitis, Breast feeding, PDA. (DOCX) [file pone.0313035.s001.docx]

Supporting Table 1. Subgroup analysis for the association between HDP and NEC≥II, stratified by GA,BW ,birth weight by gestational age,number of fetuses and place of delivery.

| Subgroup | No-HDP | HDP | Unadjusted OR (95% CI) | Adjusted OR (95% CI) |
| --- | --- | --- | --- | --- |
| Stratified by GA (wks) |  |  |  |  |
| <28,n/N (%) | 289/4485(6.4) | 30/466(6.4) | 1.00(0.66,1.45) | 1.06(0.62,1.71)^a^ |
| ≥28,n/N (%) | 902/17771(5.0) | 253/4938(5.1) | 1.01(0.87,1.16) | 0.83(0.63,1.00)^a^ |
| Stratified by BW (g) |  |  |  |  |
| ＜1000,n/N (%) | 226/3348(6.8) | 100/1406(7.1) | 1.06（0.83,1.35） | 1.09(0.74,1.59)^b^ |
| 1000-1500,n/N (%) | 668/11780(5.7) | 159/3261(4.9) | 0.85(0.71,1.02) | 0.86(0.68,1.09)^b^ |
| ≥1500,n/N (%) | 297/7128(4.2) | 24/737(3.3) | 0.77(0.49,1.16) | 1.04(0.63,1.64)^b^ |
| Stratified by Birth Weight by Gestational Age |  |  |  |  |
| SGA,n/N (%) | 48/766(6.3) | 104/1353(7.7) | 1.25(0.88,1.79) | 1.17(0.74,1.89)^c^ |
| AGA,n/N (%) | 1143/21490(5.3) | 179/4051(4.4) | 0.82(0.70,0.96) | 0.84(0.68,1.02)^c^ |
| Stratified by Birth Weight by Gestational Age |  |  |  |  |
| Singleton,n/N (%) | 830/15760(5.3) | 239/4624(5.2) | 0.98(0.84,1.13) | 0.83(0.66,1.03)^d^ |
| Twin/ Multiple,n/N (%) | 361/6496(5.6) | 44/780(5.6) | 1.02(0.73,1.39) | 1.02(0.67,1.49)^d^ |
| Inborn or outborn |  |  |  |  |
| Inborn,n/N (%) | 704/14516(4.8) | 183/3832(4.8) | 0.98(0.83,1.16) | 0.84(0.68,1.04)^d^ |
| Outborn,n/N (%) | 487/7740(6.3) | 100/1572(6.4) | 1.01(0.81,1.26) | 0.99(0.61,1.55)^d^ |

^a^ adjusted for Birth weight, Antenatal steroids, MgSO4, Chorioamnionitis, Breast feeding, PDA,.

^b^ adjusted for Gestational age, Antenatal steroids, MgSO4, Chorioamnionitis, Breast feeding, PDA.

^c^ adjusted for Antenatal steroids, MgSO4, Chorioamnionitis, Breast feeding, PDA.

d adjusted for Birth weight, Gestational age,Antenatal steroids, MgSO4, Chorioamnionitis, Breast feeding, PDA
