# Supplementary material for: Association of hypertensive disorder of pregnancy with necrotizing enterocolitis in very preterm infants: A retrospective cohort study
Source: PLoS One. 2024 Nov 11;19(11):e0313035. doi: 10.1371/journal.pone.0313035 (PMC11554212; doi:10.1371/journal.pone.0313035)
Supplement: Attachment — Submitted filename: Response to Reviewers.docx [file pone.0313035.s002.docx]

Dear editors and reviewers,

Thank you for your email and the reviewers' comments regarding our manuscript "Association of hypertensive disorder of pregnancy with necrotizing enterocolitis in very preterm infants: A retrospective cohort study" (PONE-D-24-12553). We have carefully considered the feedback and made the necessary revisions to our manuscript. Below, we address each point raised by the reviewers and the editor.

**Reviewer #1 Comments:**

**1. Add the description about the missing data of NEC (L177-178):**

**Response:** We have added a description about the missing data on NEC cases.

**original sentence**” After excluding 255 cases with multiple anomalies and 349 cases with missing maternal HDP data, 27,660 infants were analyzed.”

**revised sentence**” As there were no cases of NEC missing, after excluding 255 cases with multiple anomalies and 349 cases with missing maternal HDP data, 27,660 infants were analyzed.” (L177-178)

**Reviewer #2 Comments:**

**1.Change the Word "Conference" (L172):**

**Response:** We have corrected the spelling error in 'confidence.' Thank you for pointing it out.

**2. Clarify the discrepancy between results and discussion regarding the correlation between maternal HDP and the occurrence of NEC stages II or III.**

**Response:** We have clarified the discussion to align with the results, indicating no significant correlation between maternal HDP and NEC stages II or III after adjustments.

**original sentence**” Our study revealed that VPIs born to mothers with HDP had a lower risk of developing stage II or III NEC.”

**revised sentence**” Our study found no significant association between maternal HDP and the occurrence of stages II or III NEC in VPIs.” (L245-248)

**3. If, as the severity of NEC increases, the impact of HDP on NEC becomes more pronounced, why do you think HDP/preeclampsia/eclampsia didn't protect the VPIs against SIP?**

**Response:** We find exploring the relationship between HDP and SIP to be an intriguing topic worthy of further investigation. The evidence from our study is insufficient to support a protective role of HDP against SIP. Given that SIP and NEC involve different pathophysiological mechanisms and factors influencing their occurrence, exploring the association of HDP with SIP would require a new dedicated correlational study for verification.

**Journal Requirements:**

1. Manuscript Formatting:

**Response:** We have ensured that our manuscript follows the PLOS ONE style templates.

2. ORCID ID:

**Response:** We have validated the corresponding author's ORCID ID in the Editorial Manager.

3.Data Availability:

**Response:** The data underlying our findings are sourced from the Chinese Neonatal Network (CHNN) that we do not have the legal right to distribute. However, we provide some details to assist other researchers in accessing these data.

4.Reference List:

**Response:** We have reviewed our reference list to ensure completeness and correctness. Our references do not include any retracted papers.

We believe these revisions have strengthened our manuscript, and we hope it now meets the criteria for publication in PLOS ONE. We look forward to your positive response.

Kind regards,

Yu Hu
